# Blockage of interleukin-1β with canakinumab in patients with Covid-19

**DOI:** 10.1038/s41598-020-78492-y

**Published:** 2020-12-11

**Authors:** Lorenza Landi, Claudia Ravaglia, Emanuele Russo, Pierluigi Cataleta, Maurizio Fusari, Andrea Boschi, Diana Giannarelli, Francesca Facondini, Ilaria Valentini, Ilaria Panzini, Luigi Lazzari-Agli, Paolo Bassi, Elisa Marchionni, Rossella Romagnoli, Raffaella De Giovanni, Marina Assirelli, Federica Baldazzi, Fabio Pieraccini, Giovanna Rametta, Lucia Rossi, Luca Santini, Ivana Valenti, Federico Cappuzzo

**Affiliations:** 1 Medical Oncology 2, Istituto Nazionale Tumori Regina Elena, Via Elio Chianesi 53, 00144 Rome, Italy; 2 Pulmonology Unit, Azienda Unità Sanitaria Locale della Romagna, Forlì, Italy; 3 Anesthesia and Intensive Care Unit, Azienda Unità Sanitaria Locale della Romagna, Cesena, Italy; 4 Rheumatology Unit, Department of Internal Medicine, Azienda Unità Sanitaria Locale della Romagna, Ravenna, Italy; 5 Anesthesia and Intensive Care Unit, Azienda Unità Sanitaria Locale della Romagna, Ravenna, Italy; 6 Infectious Disease Unit, Azienda Unità Sanitaria Locale della Romagna, Rimini, Italy; 7 Anesthesia and Intensive Care Unit, Azienda Unità Sanitaria Locale della Romagna, Rimini, Italy; 8 Pulmonology Unit, Azienda Unità Sanitaria Locale della Romagna, Rimini, Italy; 9 Clinical Research Unit, Azienda Unità Sanitaria Locale della Romagna, Rimini, Italy; 10 Infectious Disease Unit, Azienda Unità Sanitaria Locale della Romagna, Ravenna, Italy; 11 Biostatistical Unit - National Cancer Institute Regina Elena, IRCCS, Rome, Italy; 12 Department of Internal Medicine, Azienda Unità Sanitaria Locale della Romagna, Ravenna, Italy; 13 Department of Internal Medicine, Azienda Unità Sanitaria Locale della Romagna, Rimini, Italy; 14 Pharmacy Unit, Azienda Unità Sanitaria Locale della Romagna, Forlì, Italy; 15 Azienda Unità Sanitaria Locale della Romagna, Rimini, Italy; 16 Pharmacy Unit, Azienda Unità Sanitaria Locale della Romagna, Rimini, Italy; 17 Pharmacy Unit, Azienda Unità Sanitaria Locale della Romagna, Ravenna, Italy; 18 Emergency Unit, Azienda Unità Sanitaria Locale della Romagna, Lugo, Italy

**Keywords:** Diseases, Health care, Medical research

## Abstract

There is the urgent need to study the effects of immunomodulating agents as therapy for Covid-19. An observational, cohort, prospective study with 30 days of observation was carried out to assess clinical outcomes in 88 patients hospitalized for Covid-19 pneumonia and treated with canakinumab (300 mg sc). Median time from diagnosis of Covid-19 by viral swab to administration of canakinumab was 7.5 days (range 0–30, IQR 4–11). Median PaO_2_/FiO_2_ increased from 160 (range 53–409, IQR 122–210) at baseline to 237 (range 72–533, IQR 158–331) at day 7 after treatment with canakinumab (*p* < 0.0001). Improvement of oxygen support category was observed in 61.4% of cases. Median duration of hospitalization following administration of canakinumab was 6 days (range 0–30, IQR 4–11). At 7 days, 58% of patients had been discharged and 12 (13.6%) had died. Significant differences between baseline and 7 days were observed for absolute lymphocyte counts (mean 0.60 vs 1.11 × 10^9^/L, respectively, *p* < 0.0001) and C-reactive protein (mean 31.5 vs 5.8 mg/L, respectively, *p* < 0.0001).Overall survival at 1 month was 79.5% (95% CI 68.7–90.3). Oxygen-support requirements improved and overall mortality was 13.6%. Confirmation of the efficacy of canakinumab for Covid-19 warrants further study in randomized controlled trials.

## Introduction

Severe acute respiratory coronavirus 2 (SARS-CoV-2) infection has created a global pandemic, with almost 9 million cases of Covid-19 and nearly 500,000 deaths as of June 22, 2020^1^. While rates of mortality vary widely in different countries, massive number of individuals needing hospitalization have overwhelmed intensive care units (ICUs) in many areas, including the most heavily affected regions in Italy^2^. Effective therapies are thus urgently needed to treat patients and minimize the need for hospitalization and potential admission to the ICU, which is associated with much higher rates of reported mortality^3^. At present, despite the large number of treatments under study, management remains largely limited to supportive care and respiratory support; additional agents such as antibiotics, antiretrovirals, and antiparasitic agents have been used on an empirical basis.

Several markers of inflammation such as C-reactive protein (CRP) and IL-6 are significantly increased in patients who die of disease compared to discharged patients^4^. Increased levels of CRP are also seen in elderly patients, who are more likely to succumb to severe disease^5^. In this regard, it is now becoming clear that the inflammatory response plays a key role in development of severe pneumonia and respiratory distress in Covid-19^6,7^. In patients with severe disease, a hyperinflammatory response and cytokine profile similar to secondary hemophagocytic lymphohistiocytosis has been observed that involves increases in IL-2, IL-7, granulocyte-colony stimulating factor, and tumor necrosis factor-α^8^. This leads to a so-called cytokine storm observed in a subset of severe patients with release of IL-1β, IL-6, IL-18, and interferon^6^. Moreover, it has been reported that infection of cells in the lower respiratory tract by SARS-CoV-2 can lead to severe acute respiratory syndrome with the subsequent consequent release of additional pro-inflammatory cytokines, including IL-1β^9^. Thus, many pro-inflammatory cytokines are now being implicated in the inflammatory response to Covid-19.

The potentially aberrant cytokine response in Covid-19 raises the possibility that the cytokine storm might be controlled by directly targeting key cytokines using existing immunomodulatory agents, thereby dampening the hyperinflammatory response^10,11^. Preliminary studies have been recently published for tocilizumab^12,13^, a monoclonal antibody that specifically targets IL-6 and anakinra^14^, an IL-1 receptor antagonist that blocks the activity of IL-1α and IL-1β, with promising results for both agents.

It is thus of immediate interest to clinically study the effects of additional immunomodulating agents as therapy for Covid-19. IL-1β is a pro-inflammatory cytokine that mediates not only immune responses during infection and inflammation, but it also has a role in acute and chronic autoinflammatory diseases^15^. Canakinumab is a fully human IgG monoclonal antibody that specifically targets IL-1β^16^. In the US and EU, it is approved for use in several autoinflammatory syndromes (periodic fevers, Still’s disease, and gouty arthritis). In addition, canakinumab is being increasingly studied for a potential role in treatment of lung cancer, and has been studied for cardiovascular protection and as anti-inflammatory therapy for atherosclerotic disease^17–19^. Based on these premises, we assessed clinical outcomes in a cohort of patients who were hospitalized for Covid-19 and treated with canakinumab.

## Results

### Baseline characteristics

**Figure 1:**
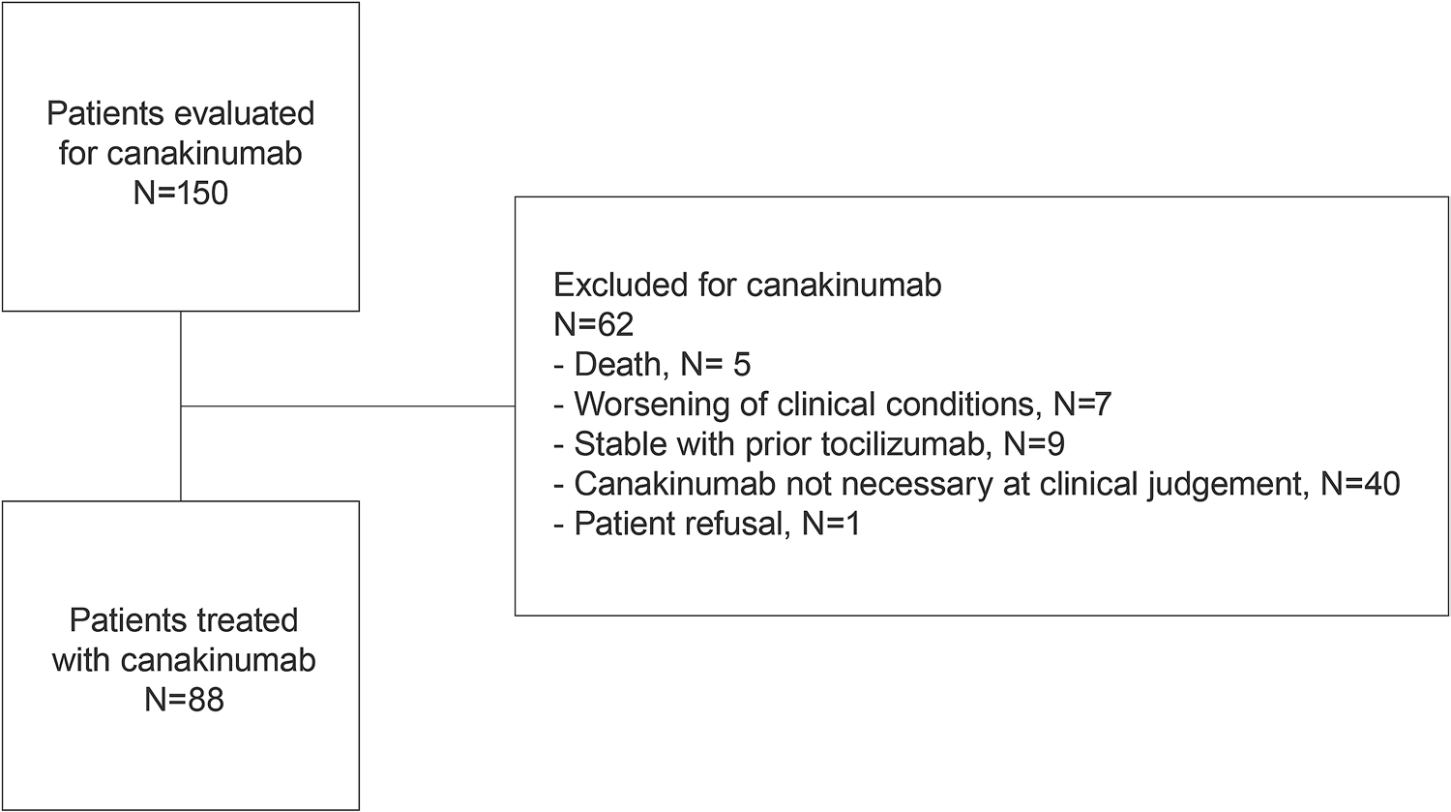
Study flow.

From March 26, 2020 to April 14, 2020 canakinumab was requested for 150 patients. It was approved for use in 88 cases and was given at a dose of 300 mg subcutaneously. The study flow chart is shown in Fig. 1 along with the reasons for exclusion in this cohort.

Table 1 details the demographic and clinical characteristics of the cohort. Median age was 67 years, and 96.6% were ≥ 50 years. Most patients had > 1 comorbidity, and 36.3% had ≥ 2 comorbidities. The most common comorbidity was cardiovascular disease, which was seen in more than one-half of patients. Eight patients had an autoimmune disease (7 cases of thyroiditis and one case of ankylosing spondylitis). The majority of patients were receiving low-flow oxygen at baseline. Most patients had also received previous therapy for Covid-19. Median time from diagnosis of Covid-19 by viral swab to administration of canakinumab was 7.5 days (range 0–30, IQR 4–11).

**Table 1:** Demographic and clinical characteristics of patients at baseline.

	Total
**Total patients, N (%)**	88 (100)
**Age, median (IQR)—years**	67 (58–73)
**Age, N (%)**	
< 50 years	3 (3.4)
50–70 years	55 (62.5)
> 70 years	30 (34.1)
**Male sex, N (%)**	67 (76.1)
**BMI (kg/m**^**2**^**), N (%)**	
< 35	20 (22.7)
≥ 35	6 (6.8)
Unknown	62 (70.5)
**Smoking, N (%)**	
Never smokers	13 (14.8)
Past/current smokers	18 (20.5)
Unknown	57 (64.8)
**Number of comorbidities, N (%)**	
0	24 (27.3)
1	32 (36.4)
2	23 (26.1)
> 2	9 (10.2)
**Comorbidities, N (%)**	
Cardiovascular disease	49 (55.7)
COPD	6 (6.8)
Diabetes mellitus	12 (13.6)
Autoimmune disease	8 (9.1)
Cancer	9 (10.2)
**Oxygen support category, N (%)**	
Ambient air	8 (9.1)
Low-flow oxygen	68 (77.3)
Non-invasive positive pressure ventilation	10 (11.4)
Invasive ventilation	1 (1.1)
Unknown	1 (1.1)
**PaO**_**2**_**/FiO**_**2**_**, N (%)**	
> 300	4 (4.5)
200 to < 300	19 (21.6)
100 to < 200	33 (37.5)
< 100	9 (10.2)
Unknown	23 (26.1)
**Median PaO**_**2**_**/FiO**_**2**_**, (range) (IQR)**	160 (53–409) (122–210)
**Lymphocyte count, N (%)**	
> 1.0 × 10^9^/L	16 (18.2)
0.8 × 10^9^/L to 1.0 × 10^9^/L	9 (10.2)
0.5 × 10^9^/L to 0.8 × 10^9^/L	29 (33.0)
< 0.5 × 10^9^/L	29 (33.0)
Unknown	5 (5.7)
**Median D-dimer, (range), (IQR)**	2071 (85–35,000) (714–6227)
**Previous therapies for Covid-19, N (%)**	
Darunavir + ritonavir	81 (92.0)
Hydroxychloroquine	83 (94.3)
High-dose steroids	76 (86.4)
Tocilizumab	8 (9.1)

*IQR* interquartile range; *BMI* body mass index; *COPD* chronic obstructive pulmonary disorder.

### Clinical improvement following treatment with canakinumab

Changes in in PaO_2_/FiO_2_ at day 7 following treatment with canakinumab were available for 45 patients (Table 2). At baseline, median PaO_2_/FiO_2_ was 160 (range 53–409, IQR 122–210), which increased to a median of 237 (range 72–533, IQR 158–331) at day 7 after treatment with canakinumab (*p* < 0.0001, Wilcoxon test). A total of 82.2% of patients showed an increase in PaO_2_/FiO_2_, while 17.8% showed a reduced PaO_2_/FiO_2_ ratio.

**Table 2:** Changes in PaO_2_/FiO_2_ at day 7 following treatment with canakinumab (300 mg sc).

	N (%)
**No. with PaO**_**2**_**/FiO**_**2**_ **data**	45 (100)
**Increase in PaO**_**2**_**/FiO**_**2**_	37 (82.2)
0–20%	7 (15.6)
21–50%	13 (28.9)
51–100%	6 (13.3)
> 100%	11 (24.4)
**Reduction in PaO**_**2**_**/FiO**_**2**_	8 (17.8)
0–20%	2 (4.4)
21–50%	4 (8.9)
51–100%	2 (4.4)
> 100%	0 (0)

Outcomes according to oxygen support category at baseline and at 7 days after receiving canakinumab are detailed in Table 3. Overall, improvement of oxygen support category was observed in 61.4% of cases. Improvement was observed in 50% patients on non-invasive support at baseline, in 61.8% of patients on low-flow oxygen at baseline, and in 87.5% of patients receiving only ambient air.

**Table 3:** Outcomes according to oxygen-support category.

	Oxygen-support group at baseline N (%)*			
	Invasive 1 (100)	Non-invasive 10 (100)	Low-flow oxygen 68 (100)	Ambient air 8 (100)
**Oxygen-support group after therapy**				
Death	0	2 (20)	8 (11.8)	1 (12.5)
Invasive	1 (100)	3 (30)	8 (11.8)	0
Non-Invasive	0	0	3 (4.4)	0
Low-flow oxygen	0	1 (10)	7 (10.3)	0
Ambient air	0	0	2 (2.9)	0
Discharged	0	4 (40)	40 (58.8)	7 (87.5)
**Improvement**	**0**	**5 (50)**	**42 (61.8)**	**7 (87.5)**

*In one patient information was not available at baseline.

Median duration of hospitalization in the entire population was 24 days (95% CI 16–32). Median duration of hospitalization following administration of canakinumab was 6 days (range 0–30, IQR 4–11). At 7 days, 58% of patients had been discharged and 12 had died, while the remaining cases had either been transferred to another hospital or had recovered but were still hospitalized (Table 4). Among the 21 women, 17 (81.0%) improved compared to 40 of 67 (59.7%) male patients (*p* = 0.08). Overall, 18 of the 21 women (85.7%) were discharged compared to 33 of the 67 men (49.3%; *p* = 0.003). All 8 patients with autoimmune diseases recovered and were discharged.

**Table 4:** Hospitalization status at day 7.

	N (%) 88 (100)	95% CI
**Hospitalization status**		
Discharged	51 (58.0%)	(47.0–68.4)
Transferred	17 (19.3%)	(11.7–29.1)
Recovered*	8 (9.1%)	(4.0–17.1)
Deceased	12 (13.6%)	(7.2–22.0)

*Clinical status improved, but still hospitalized. CI, confidence interval.

We also analyzed whether canakinumab given earlier or later in the course of disease. The median time between hospitalization and administration of canakimumab was 7 days. Among the 46 patients who received canakimumab within 7 days from hospitalization, 33 (71.1%) showed improvement at 7 days, 3 (6.5%) were stable, and 10 (21.7%) worsened. Among the 42 patients who received canakimumab after 7 days from hospitalization, 24 (57.1%) had improvement at 7 days, 1 (2.4%) was stable, and 17 (40.5%) worsened. There was no significant difference in clinical improvement between those receiving canakimumab at times < 7 days or after that time (*p* = 0.13). Similarly, there was no significant difference in mortality between these two subgroups (*p* = 0.77).

### Laboratory parameters

Regarding laboratory parameters, the only significant differences between baseline values and at 7 ± 2 days were observed for mean absolute lymphocyte counts (0.60 vs 1.11 × 10^9^/L, respectively, *p* < 0.0001) and CRP (31.5 vs 5.8 mg/L, respectively, *p* < 0.0001).

### Mortality

**Figure 2:**
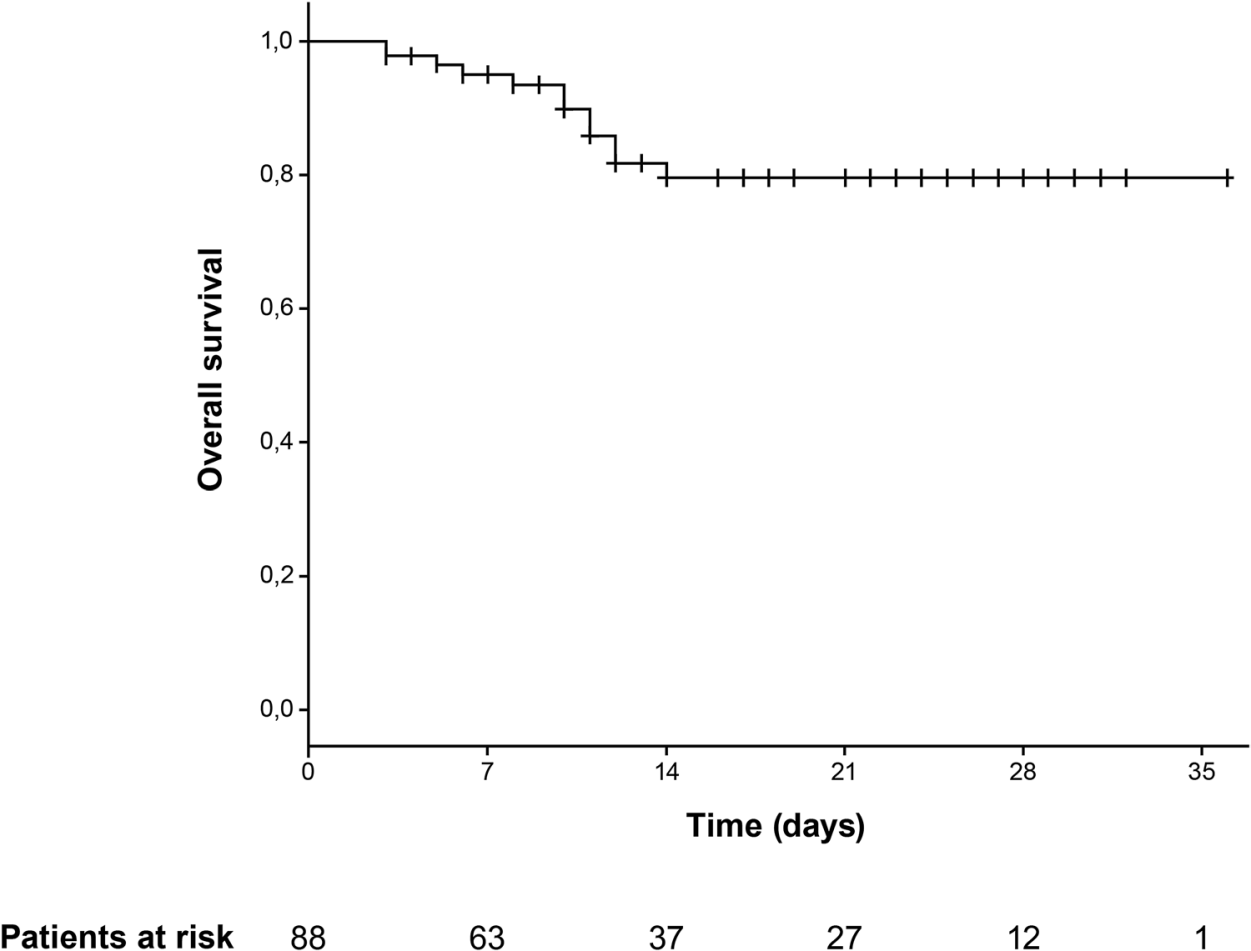
Kaplan–Meier analysis of overall survival.

Among the 12 patients who died (13.6%), median time to death was 10 days (range 3–14, IQR 6–12). The median age of these patients was 77 years (range 54–86, IQR 68–81). In Kaplan–Meier analysis, overall survival at 1 month was 79.5% (95% CI 68.7–90.3; Fig. 2). Median time from diagnosis of Covid-19 by viral swab to administration of canakinumab in patients who died was 10 days (range 1–17, IQR 6–12) and was not significantly different from the entire cohort as reported above. Of the 12 patients who died, 5 had clinically significant comorbidities. These included one patient with Parkinson’s disease, one with advanced Alzheimer’s dementia, one with cognitive impairment related to primary epilepsy, and two patients with prior hemorrhagic stroke.

### Patients receiving previous tocilizumab

Eight patients had received previous treatment with tocilizumab during hospitalization (8 mg/kg body weight), and were included in another clinical study evaluating the efficacy of tocilizumab for Covid-19. Median age of this subset of exclusively male patients was 65 years (range 32–76). Among these patients, at 7 ± 2 days, clinical improvement was seen in 3 cases (37.5%), 2 cases were in stable clinical condition (25%), 2 had worsened (25%), and one had died (12.5%).

#### Safety

Given the urgent and overwhelming conditions at the time of study, safety endpoints were not the main objective. The most common adverse event was urinary tract infection and seen in 6 patients (all Grade 2, all with a catheter in place, and none with diabetes). There were two cases each of sepsis (both Grade 4), bacterial pneumonia related to Covid-19 (both Grade 3), and thrombocytopenia (both Grade 3). Lastly, there was one case each of hemolytic anemia (Grade 3), renal insufficiency (Grade 2), and empyema (Grade 2).

## Discussion

At present, management of patients with Covid-19 is largely based on supportive care, and many empirical treatments are given on a compassionate basis. This was also obvious in the present cohort of patients, and, before administration of canakinumab the vast majority had received a combination darunavir and ritonavir as well as hydroxychloroquine and steroids in accordance with current recommendations from the Società Italiana di Malattie Infettive e Tropicali (SIMIT) in Italy^20^. However, as clinical experience is increasing it is becoming clearer that based on the results of the large RECOVERY trial, dexamethasone appears to be beneficial in patients receiving invasive mechanical ventilation or oxygen support^21^. There is also evidence to suggest that remdesivir shortens the time to recovery in patients hospitalized for Covid-19^22^. In contrast, the use of hydroxychloroquine does not seem to improve clinical status^23^.

The current analysis presents clinical outcomes in patients with Covid-19 and receiving canakinumab, and it is clear that additional studies are needed on the efficacy of the drug in Covid-19, and especially safety. The use of canakinumab for Covid-19 has been documented in a case report of an 85-year-old man with acute respiratory distress syndrome and cardiac and renal failure^24^, and very recently, Ucciferri et al. reported on a preliminary retrospective analysis of 10 patients with Covid-19 and respiratory failure^25^. All patients recovered within 45 days, and the authors concluded that canakinumab was well tolerated, improved oxygenation, and decreased the systemic inflammatory response. Our results confirm and expand upon these findings. In particular, PaO_2_/FiO_2_ increased in 82.2% of patients, and improvement of oxygen-support category was seen in 61.3% of cases. Overall mortality was 13.6% in this group of patients after a median duration of hospitalization of 19 days. It should also be highlighted that of the 12 patients who died, 5 had clinically significant comorbidities that likely increased the severity of Covid-19. While there were no significant differences between median time of diagnosis in patients who died compared to survivors (10 days vs. 7.5 days, respectively), we still believe that it is preferable to start treatment early, even if no statistically significant difference was between those receiving canakimumab at < 7 days or > 7 days. As stated by other authors, given the lack of a specific therapy, management of patients with Covid-19 should entail best supportive care as for any respiratory disease^26^. This thus implies the early use of antimicrobials, neuraminidase inhibitors, and other agents as needed for pre-existing comorbidities.

Our findings on mortality are comparable to those reported in a recent analysis of the compassionate use of remdesivir, wherein 68% of patients improved oxygen support category and 13% of patients died^27^.In a randomized controlled trial of lopinavir + ritonavir in patients hospitalized for Covid-19, mortality at 28 days was 22%^28^. Widely variable mortality rates have been reported that depend, however, on the need for admission to the ICU and/or invasive ventilation, but in large cases series appears to be around 20–25%^29,30^. The mortality rate observed herein is also similar to that reported with the use of high-dose anakinra in patients with Covid-19 acute respiratory distress syndrome and hyperinflammation where after 1 days, 10% of patients had died. Treatment with anakinra was further associated with improvements in both overall respiratory function (21 of 29 patients, 72%) and reductions in CRP^14^. Anakinra is of interest as it works via blockade of IL-1α and IL-1β, and canakinumab through inhibition of the latter. While no clear correlations can be drawn between the clinical results with anakinra and canakinumab, the combined findings would nonetheless appear to give additional weight to the possibility that inhibition of IL-1 may have therapeutic benefits in patients with Covid-19.

The findings for CRP in that report are in agreement with the changes in laboratory parameters observed herein, where mean CRP decreased from 31.5 to 5.8 mg/mL after treatment with canakinumab. It has been reported that levels of CRP increase early and appear to positively correlate with pulmonary damage that reflects the severity of disease^31^. A meta-analysis of 1994 patients reported that an increase in CRP is present in 44.3% of cases, with a fatality rate of 5%. Another meta-analysis on 8697 patients reported that CRP was elevated in 65.9% of patients^32^. However, these analyses included all patients seeking clinical attention with varying degrees of severity, including asymptomatic cases.

Eight patients in the present cohort had received prior therapy with tocilizumab. Of note, in all cases tocilizumab was administered subcutaneously and not via an intravenous route as the latter formulation was not available. Since tocilizumab is normally administered intravenously when given for cytokine storm, this might explain the lack of efficacy observed. A small number of studies have investigated the use of tocilizumab as treatment for Covid-19. Following administration of tocilizumab in 15 patients, Luo et al. reported that rapidly decreased levels of IL-6, an event that appeared to be associated with clinical improvement^33^. Xu documented that administration of tocilizumab to 20 patients with severe Covid-19 led to recovery and discharge of all patients after a mean of 15 days^13^. In a large single center study in 100 patients with severe Covid-19 and hyperinflammatory syndrome, mortality was observed in 20% of cases receiving tocilizumab^34^. The small study by Klopfenstein et al. reported that deaths and admissions to the ICU were higher in patients treated with tocilizumab^12^, while, in contrast, Colaneri et al. found that the drug had no effects on either mortality or admission to the ICU^35^. A recent randomized trial investigating tocilizumab in 243 patients hospitalized for Covid-19 reported that it did not appear to be of benefit in preventing intubation or death^36^. In our cohort, in the 8 patients who received prior tocilizumab, clinical improvement was seen at 7 ± 2 days after administration of canakinumab in 3 patients; 2 cases were still in a stable clinical condition, while 3 had worsened and 1 had died. However, it must also be mentioned that our patients had later been administered canakinumab, which renders direct comparisons impossible.

Two cases of sepsis were observed, both of whom were receiving high doses of steroids. Sepsis is not an uncommon finding in patients with acute respiratory distress syndrome, and guidance has been issued for caring for critically-ill Covid-19 patients in this regard^37^. As prevention for atherosclerotic disease, a greater number of deaths attributed to sepsis/infection have been reported for canakinumab compared to placebo, although the overall incidence was low^38^. In the Summary of Product Characteristics, it is stated that canakinumab is associated with an increased incidence of serious infections. The risk of infections with canakinumab has also been related to older age and chronic kidney disease^38^. Notwithstanding, given the overall clinical conditions of hospitalized patients with Covid-19, in the absence of additional studies, it would be difficult to directly relate the presence of sepsis to administration of canakinumab, also considering that these patients had been heavily pretreated with a variety of diverse drugs. In this regard, it is interesting to note that bacteremia was reported in 14% of patients treated with anakinra^14^.

The limitations of our study are clear. The cohort was relatively small and follow-up was limited to 1 month, at which time final outcomes were not available for all patients. Moreover, our data set is not complete: additional information on laboratory parameters would be desirable as is data on safety and tolerability. Further study could also help to define a subset of patients in which canakinumab is more effective. Of note, virtually all patients were heavily pretreated with other agents, including tocilizumab, hydroxychloroquine, and steroids, which were common empirical therapies at the time of the emergency situation. Thus, concomitant administration of other agents, especially steroids, is a potentially confounding factor. Thus, given all these aspects, caution is mandated interpreting the findings reported herein. In our opinion, considering our clinical experience, further studies of canakinumab in patient with Covid-19 are warranted. In this regard, the Phase III CAN-COVID trial is currently recruiting patients with Covid-19-induced pneumonia and cytokine release syndrome who will be randomized to either standard of care or canakinumab plus standard of care. Diverse from the present study where patients received a 300 mg dose of the drug subcutaneously, canakinumab will be administered by infusion over 2 h with dosing based on body weight (from 450–750 mg). Canakinumab is also being investigated for its ability to reduce deterioration of cardiac and respiratory function in patients with Covid-19 and myocardial injury with heightened inflammation^39^.

## Methods

### Study design and patients

The present observational study (CANASCOV) describes outcomes in a cohort of patients with pneumonia related to Covid-19 who were admitted to hospitals belonging to AUSL Romagna, Italy, and were treated with canakinumab. The study was conducted in accordance with the relevant guidelines and regulations for clinical trial research and was approved by the Ethics committee of AULS Romagna. All patients provided written informed consent for inclusion in the study. Romagna is a geographic area located in the North-eastern area of Italy with more than 1,200,000 inhabitants. Five main hospitals are located in the area, including Ravenna, Lugo, Faenza, Rimini, and Forlì-Cesena. Starting on March 18, 2020, Novartis began accepting requests from clinicians for off-label use of canakinumab. The use of canakinumab was approved by the Agenzia Italiana del Farmaco (AIFA) through an off-label use program according to Law 94/1998 and registered on clinicaltrials.gov (NCT04348448). To submit a request, clinicians completed a specific form with demographic and disease-status information of the patient. A request was approved only for patients ≥ 18 years of age with confirmed or presumptive infection SARS-CoV-2 infection using a nasal swab test to detect viral RNA, and was not contingent upon the need for intubation or ventilation procedures. Pneumonia was diagnosed with chest X-ray or chest computed tomography. For patients not requiring intubation, details on oxygen saturation and type of ventilation was collected. Canakinumab was administered as a single subcutaneous injection of 300 mg. Supportive care was given at the clinician’s discretion. Follow-up continued for at least 21 days after treatment with canakinumab or until discharge or death.

There was no prespecified study endpoint. Data on oxygen-support requirements, adverse events, and laboratory values, including lymphocytes, CRP, D-Dimer, lactate dehydrogenase (LDH), IL-6, serum creatinine, alanine aminotransferase (ALT), and aspartate aminotransferase (AST), were collected on day 1 and on day 7 after canakinumab administration. Additionally, information on changes in needs for oxygen support (i.e. low-flow oxygen, high-flow oxygen, invasive ventilation) was collected as well as hospital discharge and death. Focus was placed on two main outcomes: i) respiratory improvement [changes in the arterial partial pressure of oxygen/fraction of inspired oxygen (PaO_2_/FIO_2_) ratio following canakinumab administration and day 7); ii) clinical improvement, as recommended by the WHO R&D Blueprint Group and defined by discharge from hospital, a decrease of at least 2 points from baseline on a modified ordinal scale, or both. Safety was evaluated through documentation of main adverse events and their severity using NCI-CTCAE version 4.0.

### Statistical analysis

The cohort included all patients who received a single dose of canakinumab from March 26 to April 14, 2020, and for whom clinical data for at least 1 subsequent day were available. Clinical improvement, discharge rate, and mortality were considered as main outcomes, while mortality was investigated by the Kaplan–Meier approach. For calculation of the median value of hospitalization, deceased patients were considered as never discharged (i.e. their stay was assumed longer than the longest follow-up). Characteristics prior to treatment with canakinumab and main outcomes were evaluated with a chi-square test. Differences between baseline and after 7 days were assessed using the Wilcoxon signed rank test. Results are reported as point estimates and 95% confidence intervals when referring to rates and as median, minimum and maximum values, and interquartile range (IQR). All analyses were carried out with IBM SPSS software, version 23.0.

## Data Availability

The datasets generated during and/or analyzed during the current study are available from the corresponding author on reasonable request.
